# Extracellular vesicle–derived miR-141, miR-10b, miR-550a and miR-16 define a circulating signature in prostate cancer

**DOI:** 10.1007/s11845-026-04482-0

**Published:** 2026-06-12

**Authors:** Emine Yagci, Hulyam Kurt, Ata Ozen, Cansu Ozbayer, Selma Metintas

**Affiliations:** 1 Faculty of Medicine, Department of Medical Biology, Kutahya Health Sciences University, Kutahya, Türkiye; 2 Faculty of Medicine, Department of Medical Biology, Eskisehir Osmangazi University, Eskisehir, Türkiye; 3 Faculty of Medicine, Department of Urology, Eskisehir Osmangazi University, Eskisehir, Türkiye; 4 Faculty of Medicine, Department of Public Health, Lung and Pleural Cancers Research and Clinical Center, Eskisehir Osmangazi University, Eskisehir, Türkiye

**Keywords:** Extracellular vesicles, Prostate cancer, Liquid biopsy, miR-10b, miR-141, miR-550a

## Abstract

**Background:**

Prostate cancer (PCa) diagnosis relies heavily on PSA screening, which suffers from low specificity, leading to unnecessary biopsies and overdiagnosis. Distinguishing PCa from benign prostatic hyperplasia (BPH) remains a significant clinical challenge. Extracellular vesicles (EVs) protect molecular cargo, such as microRNAs (miRNAs), from degradation and reflect the physiological state of their parental cells. Given their stability and accessibility in biological fluids, EV-associated miRNAs represent ideal liquid biopsy candidates to overcome the limitations of current diagnostic markers and improve risk stratification.

**Aims:**

This study investigated the potential of a specific plasma EV-derived miRNA panel (miR-16, miR-141, miR-10b, miR-373, miR-409, miR-34a, miR-205, miR-145, and miR-550a) to differentiate between healthy controls, BPH, localized PCa (LPCa), and metastatic PCa (MPCa).

**Methods:**

Plasma EVs were isolated using a precipitation-based method, followed by total RNA extraction. EV-derived miRNA expression profiles were quantified via qRT-PCR. Statistical analyses were performed to compare expression levels across clinical groups and to assess their diagnostic potential.

**Results:**

Significant differential expression was observed for EV-derived miR-141, miR-10b, and miR-550a. miR-141 was significantly downregulated in BPH and LPCa compared to healthy controls (*p* < 0.05). Notably, EV-derived miR-10b and miR-550a levels were significantly lower in the MPCa group compared to BPH. When comparing all PCa cases (LPCa+MPCa) against non-cancerous groups (BPH+Control), EV-derived miR-10b and miR-550a were significantly downregulated, while miR-16 showed upregulation in the cancer cohort.

**Conclusion:**

Our findings demonstrate that specific plasma EV-derived miRNA signatures can distinguish prostate pathologies and disease stages. These results suggest that EV-derived miR-10b, miR-141, miR-16 and miR-550a hold promise as non-invasive biomarkers for differentiating PCa from BPH, potentially reducing overdiagnosis and informing clinical management.

**Graphical Abstract:**

**Figure:**
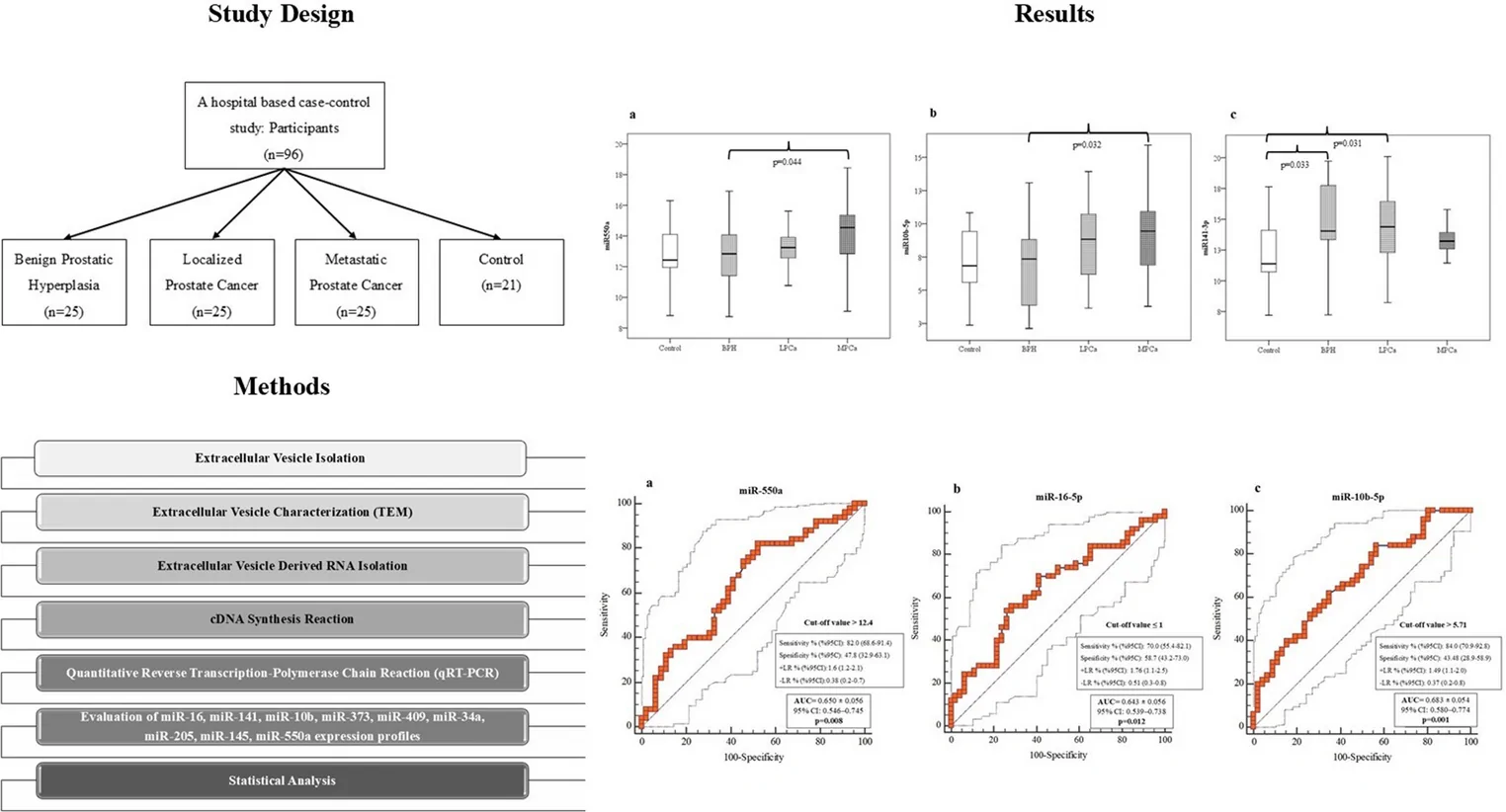

## Introduction

Prostate cancer (PCa) is one of the most prevalent malignancies among men worldwide and remains a leading cause of cancer-related mortality. In contrast, benign prostatic hyperplasia (BPH) is a common non-malignant condition characterized by urinary tract symptoms that can mimic early-stage PCa. While PSA screening has significantly improved early diagnosis, its limited specificity often results in unnecessary biopsies and an increased clinical burden. Consequently, there is a need for more specific biomarkers to accurately differentiate PCa from BPH and healthy conditions [1, 2].

Extracellular vesicles (EVs) are nanoscale membrane-bound vesicles secreted into biological fluids by various cell types. By encapsulating proteins, lipids, DNA, and diverse RNA species, including microRNAs (miRNAs), they facilitate protected transport and intercellular communication. Because their molecular cargo reflects the physiological and pathological status of their donor cells, EVs have emerged as a promising source of biomarkers within liquid biopsies. Notably, EV-derived miRNAs exhibit greater stability than their free-circulating counterparts and may significantly enhance diagnostic specificity for various malignancies, including prostate cancer (PCa) [1]. In cancer, they are involved in angiogenesis, cell migration and proliferation, immune suppression, escape from immune surveillance, and induction of metastasis [3].

MicroRNAs (miRNAs) are a class of small non-coding RNAs that modulate gene expression at the post-transcriptional level by either inducing mRNA degradation or suppressing protein translation [4]. Through these mechanisms, they play pivotal roles in oncogenic pathways, tumor suppression, apoptosis, metastasis, and therapeutic resistance. Specific circulating miRNAs, including miR-144, miR-375, miR-21, and miR-107, have been frequently identified across various cohorts as potential biomarkers for prostate cancer (PCa). However, their diagnostic performance and consistency remain inconsistent across different studies and biological matrices. Furthermore, many of these candidates have yet to be rigorously validated within EV-rich samples [5]. Given the diagnostic limitations of current PSA screening, identifying specific miRNA signatures encapsulated within EVs offers a promising and robust strategy to accurately differentiate prostate cancer from benign conditions through liquid biopsy.

Despite the expanding repertoire of candidate miRNAs implicated in prostate tumorigenesis, gaps persist in our understanding of how specific EV-associated miRNAs perform in differentiating PCa from benign prostatic hyperplasia (BPH). Building upon documented evidence of dysregulation in prostate pathology and their functional involvement in cancer-related pathways, such as EMT, cell cycle regulation, and metastasis, we selected a targeted panel of nine EV-derived miRNAs representing both oncogenic and tumor-suppressive roles. For instance, miR-16 has been linked to PCa progression, while miR-205 is known to modulate epithelial-mesenchymal transition and invasiveness within prostate cells [6–8].

The clinical application of circulating or EV-derived miRNAs for prostate cancer diagnosis remains constrained. Biological heterogeneity arising from varying disease stages, the influence of the tumor microenvironment, and patient-specific molecular profiles can significantly alter miRNA secretion patterns. Furthermore, the overlapping molecular signaling pathways between BPH and early-stage PCa complicate differential diagnosis. Given that prostate cancer exhibits a slow yet heterogeneous progression, early molecular differentiation between malignant and benign conditions is vital for reducing overtreatment, enhancing risk stratification, and advancing personalized medicine. Consequently, identifying EV-derived miRNA signatures with both diagnostic and biological significance could substantially improve the clinical management of prostate diseases [9, 10].

The specific miRNA panel used in our study (miR-16, -34a, -145, -205, -373, -409, -10b, -141 and − 550a) was strategically designed to encompass three critical axes of prostate cancer progression: osteomimicry, PI3K/AKT signaling, and EMT plasticity. First, given that bone is the dominant site of metastasis in PCa, we targeted miR-141 and miR-409 due to their roles in regulating osteomimicry a process by which tumor cells acquire bone-like features to survive in a skeletal environment, a mechanism recently highlighted as a key diagnostic and therapeutic target in bone metastasis [11, 12].

Secondly, miR-34a, miR-145, and miR-16 were included in the panel because they are tumor suppressors that antagonize the persistently active PI3K/AKT/mTOR signaling pathway in cancer cells. The expression levels of these molecules are critical indicators reflecting the cell’s regulatory capacity to inhibit oncogenic growth signals and the extent to which this control mechanism has been lost [13, 14]. Finally, miR-205, miR-373, and miR-10b were selected to assess epithelial-mesenchymal transition (EMT); these microRNAs are potential candidates that may contribute to the tumor’s transition to the metastatic process [15, 16]. By profiling this multifunctional panel, we aimed to capture a comprehensive snapshot of the systemic changes accompanying prostate carcinogenesis.

To address these challenges, the present study analyzed EV-associated miRNA expression in the plasma of patients with BPH, localized PCa, and metastatic PCa, alongside healthy controls. Our objective was to characterize distinct miRNA expression patterns and identify signatures with potential utility in non-invasively distinguishing PCa from both BPH and healthy states.

## Methods

### Patient recruitment and sample collection

Following clearance from the Eskişehir Osmangazi University Clinical Research Ethics Committee decisions numbered 2019/42, this research was conducted.

The study included 25 patients with benign prostatic hyperplasia (BPH), 25 patients with localized prostate cancer (LPCa), 25 patients with metastatic prostate cancer (MPCa) and, 21 healthy control individuals who applied to the Department of Urology, Eskişehir Osmangazi University, Medical Faculty, Türkiye. After the patients were diagnosed, 4 ml of blood was taken from the patient and control individuals and plasma stored at -80 °C until used in EV isolation.

### Extracellular vesicle isolation

In our study, Extracellular vesicle Isolation Kit (Invitrogen by Thermo Fisher Scientific Corporation, Carlsbad, California, USA) was used for EV isolation.

The total EV isolation reagent from plasma contained in the EV Isolation Kit binds water molecules, separating less soluble components such as EVs from the solution. 500 µL plasma was used in our study as the starting material for EV isolation. To extract platelets, subcellular fragments, and bigger microparticles from plasma, differential centrifugation must be used, first at low speed (2000 g) and subsequently at high speed (10000 g). Isolation was carried out in accordance with the kit protocol.

### Extracellular vesicle characterization

The EVs were characterized by transmission electron microscopy (TEM) to assess morphological features and size distribution. TEM analysis was conducted at the Eskişehir Osmangazi University Central Research Laboratory Application and Research Center. Morphological observations indicated that EVs in the control group generally exhibited a regular, circular appearance. In contrast, EVs from the BPH and prostate cancer groups displayed a more diverse morphology, including oval shapes and less regular outlines. EV diameters were measured using scale-calibrated images and ImageJ software (National Institutes of Health, Bethesda, MD, USA). The identity of the isolated particles was confirmed as EVs based on their characteristic size ranges and morphological consistency with established EV profiles.

### Extracellular vesicle RNA isolation

The MagMAX™ mirVana™ Total RNA Isolation Kit (Applied Biosystems by Thermo Fisher Scientific, Vilnius, Lithuania) was used for RNA isolation from EVs, following the manufacturer’s instructions. EV samples were first treated with Proteinase K and lysed. The RNA was then bound to RNA Binding Beads and eluted. Subsequently, residual genomic DNA was removed by treatment with TURBO™ DNase, and the RNA was re-bound to the beads. Finally, the RNA was recovered using Elution Buffer. The concentration of the isolated RNA was quantified using a QFX Fluorometer (DeNovix, USA) with the Qubit RNA Assay Kit.

### cDNA synthesis reaction

A universal cDNA library was generated from isolated EV-derived miRNAs using the TaqMan™ Advanced miRNA cDNA Synthesis Kit (Applied Biosystems, CA, USA) according to the manufacturer’s instructions. Unlike traditional miRNA-specific reverse transcription (RT), this approach employs a sequence-independent modification of miRNAs. Briefly, the process involves polyadenylation of the 3’ ends, followed by the ligation of an adapter to the 5’ ends of the mature miRNAs. Subsequently, universal reverse transcription was performed using primers targeting these modified ends. To ensure sufficient template availability for subsequent analysis, a universal pre-amplification step (miR-Amp) was performed. This step uniformly increases the concentration of all cDNA species without introducing sequence-specific bias, which is critical for samples with low initial RNA yields, such as EV-derived RNA. The resulting miR-Amp products were stored at -20 °C and used as templates for targeted qRT-PCR analysis.

### Quantitative reverse transcription-polymerase chain reaction

Using the RT-qPCR mix, RT-qPCR was made for using StepOneTM Real-Time PCR (Applied Biosystems, Carlsbad, CA). RT-PCR mix consist of 5 µl of the BrightGreen miRNA qPCR Master Mix (Applied Biological Materials Inc., Canada), 2 µl of nuclease-free water, 0.5 µl of Forward and Reverse Primer, and 2 µl of cDNA under the following RT-PCR conditions: 95 °C for 10 min and 40 cycles of 95 °C for 10 s, 63 °C for 15 s, and 72 °C for 20 s and final extension 95 °C for 5 min.

miRNA RT-PCR primers of hsa-U6-2 (MPH00001), hsa-miR-16-5p (MPH02234), hsa-miR141-3p (MPH02194), hsa-miR10b-5p (MPH02074), hsa-miR373-3p (MPH02555), hsa-miR409-3p (MPH01604), hsa-miR34a-5p (MPH02500), hsa-miR205-5p (MPH02311), hsa-miR145-5p (MPH02201), hsa-miR550a (MPH01845) were obtained from the Applied Biological Materials (Richmond, BC, Canada).

### Data processing and relative quantification

The comparative threshold cycle (Ct) values were made by the StepOne Software v2.3 for endogenous control U6, hsa-miR-16-5p, hsa-miR141-3p, hsa-miR10b-5p, hsa-miR373-3p, hsa-miR409-3p, hsa-miR34a-5p, hsa-miR205-5p, hsa-miR145-5p, hsa-miR550a primers. By normalizing the ΔCt values of the samples to the Ct values of the endogenous control U6, the values were ascertained. Using the Microsoft Excel application, the comparative Ct (2^−ΔΔCt^) approach [17] was used to assess the relative miRNA expressions of the patient and control samples.

### Statistical analysis

SPSS (version 15.0) and MedCalc Statistical Software (version 19.1.16, Medcalc Software Ltd, Ostend, Belgium) were used to analyze the data. Mean ± SD, median, and min-max values were provided for the continuous data in the study. The agreement of the data with the normal distribution was checked using graphs and the Shapiro-Wilk test. Nonparametric tests were used in the analysis when the data were not normally distributed. Continuous variables were expressed as mean ± SD, median and 25th (Q1) to 75th (Q3) percentile. In terms of frequency and percentage, categorical variables were stated.

The Mann–Whitney U test was employed to compare EV-derived miRNA ΔCt values between prostate cancer groups (LPCa and MPCa) and non-cancerous groups (BPH and controls). EV-derived miRNA ΔCt and miRNA 2^−ΔΔCt^ values were compared among groups were performed using the Kruskal–Wallis test. When overall significance was detected, post hoc pairwise comparation were performed to identify inter-group differences.

Using MedCalc statistical software and receiver operator characteristics curve (ROC) analysis, the control and patient group for each biomarker were determined. The area under the curve (AUC) value in the ROC analysis was determined by calculating the cut-off value of biomarkers that gave a significant value of *p* < 0.05.

## Results

**Fig. 1:**
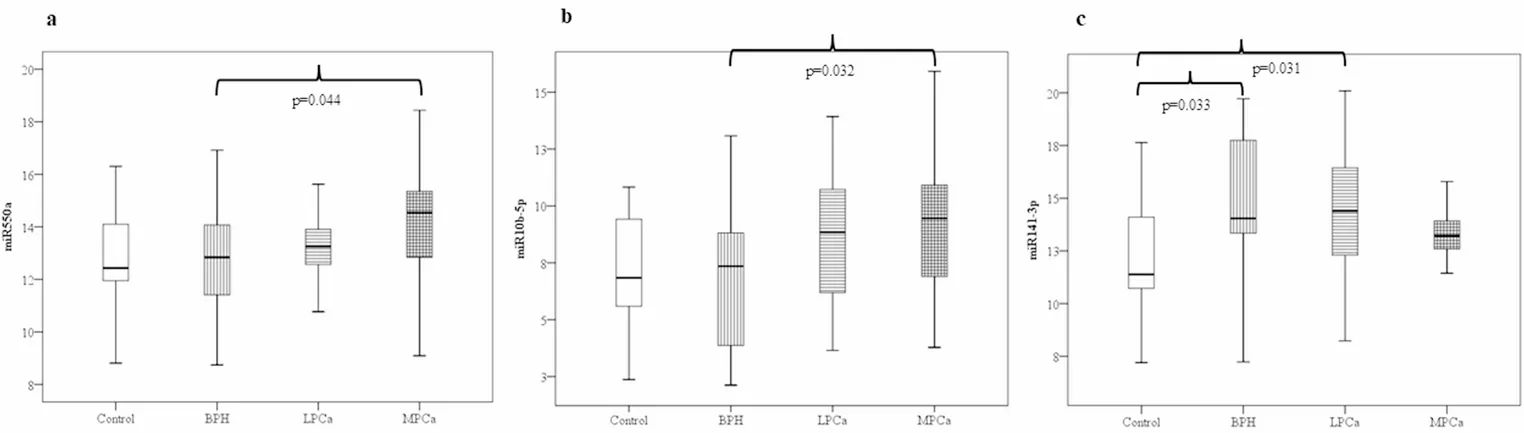
EV-derived miR-550a (**a**), miR-10b-5p (**b**), miR-141-3p (**c**) expression profiles of study groups, *Y axis; ΔCt values of miRNAs. Box plots show miR-550a (a)*,* miR-10b-5p (b)*,* miR-141-3p (c) levels across groups (Control*,* BPH*,* LPCa*,* MPCa). The median is indicated by the horizontal line*,* boxes represent the interquartile range (25th–75th percentiles)*,* and whiskers extend to the most extreme values within 1.5×IQR*

It was determined that the expressions of EV-derived miR-141-3p, miR10b-5p, miR550a were evaluated differed between the study groups (Control, BPH, LPCa, MPCa). miR-141-3p expression was down-regulated in the BPH and LPCa groups compared to the control group. miR-10b-5p and miR-550a expressions were down-regulated in the MPCa group compared to the BPH group. The comparisons of ΔCt values of miRNAs demonstrated borderline statistical significance and high variability. The box plots illustrate the median values of ΔCt values of, with interquartile ranges (IQRs), while whiskers indicate range excluding outliers (Fig. 1). However, no statistically difference was detected between the study groups in the expression levels of the other 5 miRNAs examined (mir-16, miR-373, miR-409, miR-34a, miR-205, and miR-145) (Table 1).

**Table 1:** The Comparison of EV-derived miRNA ΔCt values of BPH, LPCa, MPCa and healthy control groups

miRNA	Control (*n* = 21) Median (Q1-Q3)*	BPH (*n* = 25) Median (Q1-Q3)	LPCa (*n* = 25) Median (Q1-Q3)	MPCa (*n* = 25) Median (Q1-Q3)	p**
miR16-5p	1.03 (-0.26-2.58)	1.33 (-0.25-1.85)	-0.22 (-1.60-0.90)	0.33 (-1.30-1.96)	0.065
miR141-3p	11.38^a^ (10.47–14.35)	14.04^b^ (13.25–17.97)	14.39^b^ (12.14–17.39)	13.21 (12.34–14.12)	**0.022**
miR10b-5p	6.84 (5.48–9.61)	7.35^a^ (3.56–9.51)	8.84 (6.18–10.77)	9.45^b^ (6.75-11.00)	**0.012**
miR373-3p	11.58 (10.25–13.53)	11.17 (9.75–12.09)	11.22 (9.97–12.61)	12.40 (10.75–14.44)	0.161
miR409-3p	13.33 (11.05–15.89)	13.16 (11.52–14.18)	13.86 (11.78–16.15)	14.72 (12.77–16.56)	0.276
miR205-5p	14.97 (11.76–17.74)	16.30 (9.99–18.58)	16.44 (13.29–18.67)	15.53 (14.34–16.88)	0.872
miR34a-5p	15.04 (12.71–17.68)	17.16 (13.32–18.19)	16.85 (14.75–18.74)	16.99 (14.51–19.66)	0.276
miR145-5p	8.26 (7.11–10.07)	8.03 (6.18–9.99)	7.94 (6.47–9.60)	9.85 (7.04–11.29)	0.322
miR550a	12.43 (11.90-14.24)	12.84^a^ (11.38–14.08)	13.25 (12.50-14.14)	14.54^b^ (12.84–15.54)	**0.034**

*BPH*: Benign Prostatic Hyperplasia, *LPCa*: Localized Prostate cancer, *MPCa*: Metastatic Prostate cancer;

*Q1–Q3: 25th–75th percentiles, **: Since it did not show a normal distribution, p values were obtained using the Kruskal-Wallis test, a,b: Differences were detected between the groups indicated with different letters, Following significant Kruskal–Wallis test, pairwise group comparisons were performed. Bold: Represents statistically significant p values (*p* < 0.05).

An inverse correlation exists between ΔCt values and relative miRNA expression levels. Since ΔCt was calculated as Ct(target)-Ct(reference), higher ΔCt values indicate a lower abundance of the target miRNA, whereas lower ΔCt values reflect higher expression levels.

As a result of the comparison of expression levels between prostate cancer (LPCa and MPCa) and non-cancerous (BPH, Control) groups, it was determined that miR-10b-5p and miR-550a were down-regulated in the cancer group, while miR-16-5p was up-regulated in the cancer group. No statistical difference was observed between the prostate cancer (LPCa and MPCa) and non-cancerous (BPH, Control) groups in the expression levels of the remaining 6 miRNAs (miR-141, miR-373, miR-409, miR-34a, miR-205, and miR-145) (Table 2).

**Table 2:** EV-derived miRNA ΔCt values of prostate cancer patients (LPCa and MPCa groups) and non-cancerous groups (BPH and control groups)

miRNA	Prostate cancer (*n* = 50) Median (Q1-Q3)*	Non-cancerous (*n* = 46) Median (Q1-Q3)	p**
miR16-5p	-0.10 (-1.48-1.54)	1.25 (-0.24-1.93)	**0.016**
miR141-3p	13.68 (12.25–15.83)	13.64 (11.22–15.54)	0.337
miR10b-5p	9.40 (6.49–10.84)	6.98 (4.74–9.51)	**0.002**
miR373-3p	11.73 (10.44–13.46)	11.48 (9.91–12.43)	0.271
miR409-3p	14.15 (12.10-16.28)	13.26 (11.39–14.31)	0.082
miR205-5p	15.72 (13.52.99–18.06)	15.64 (10.79–18.13)	0.403
miR34a-5p	16.92 (14.59–19.16)	16.68 (12.95–17.94)	0.121
miR145-5p	8.44 (6.60-10.52)	8.15 (6.99–10.04)	0.641
miR550a	13.73 (12.70-15.05)	12.53 (11.64–14.09)	**0.011**

*:Median ΔCт values; Q1–Q3: 25th–75th percentiles,

**:Mann-Whitney U test. Bold: Represents statistically significant p values (*p* < 0.05)

**Fig. 2:**
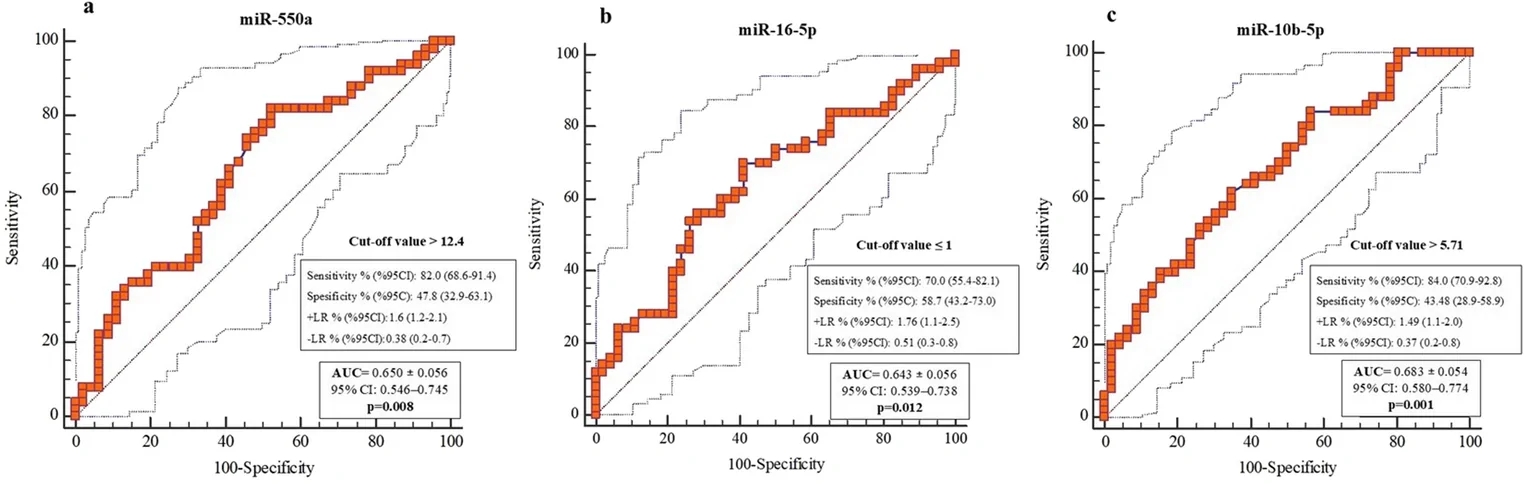
Receiver operating characteristic (ROC) curves of EV-derived miR-550a (**a**), miR-16-5p (**b**), miR-10b-5p (**c**) in prostate cancer and non-cancerous group

To assess the potential of plasma EV-derived miRNAs to differentiate between the prostate cancer (LPCa+MPCa) and non-cancer (BPH+Control) cohorts, a Receiver Operating Characteristic (ROC) curve analysis was conducted. The diagnostic performance parameters, including Area Under the Curve (AUC), 95% Confidence Intervals (CI), and p-values, are presented in Fig. 2. The ROC analysis indicates that EV-derived miR-10b-5p, miR-550a, and miR-16-5p exhibit statistically significant discriminatory power in distinguishing prostate cancer from the non-cancerous groups in our study population.

The relative expression levels (Fold Change, 2^−ΔΔCt^) of the evaluated EV-derived miRNAs across the BPH, LPCa, and MPCa groups are detailed in Table 3. Statistically significant differences in expression levels were observed among the groups for miR-10b-5p (*p* = 0.037), miR-373-3p (*p* = 0.033), and miR-550a (*p* = 0.024). Specifically, the median fold change values for these three miRNAs demonstrated a a decreasing pattern from the BPH group toward the metastatic (MPCa) stage. For instance, the median expression of miR-550a decreased from 0.98 in the BPH group to 0.30 in the MPCa group. Similarly, miR-10b-5p and miR-373-3p reached their lowest median levels in the MPCa cohort (0.18 and 0.62, respectively). No statistically significant differences were observed for the remaining miRNAs (*p* > 0.05) within these specific group comparisons.

**Table 3:** EV-derived miRNA 2^−ΔΔCt^ values of BPH, LPCa and MPCa groups

miRNA		Fold Change		
	BPH Median (Q1-Q3)	LPCa Median (Q1-Q3)	MPCa Median (Q1-Q3)	*p*
miR16-5p	0.84 (0.60–1.84)	2.44 (1.15–5.39)	1.67 (0.65–3.18)	0.060
miR141-3p	0.25 (0.02–0.41)	0.84 8(0.28–2.55)	0.52 (0.26–1.50)	0.267
miR10b-5p	0.75 (0.27–8.31)	0.27 (0.07–1.68)	0.18 (0.06–1.02)	**0.037**
miR373-3p	1.45 (0.78–3.49)	1.40 (0.58–3.04)	0.62 (0.15–1.66)	**0.033**
miR409-3p	1.22 (0.64–3.49)	0.75 (0.17-3.00)	0.42 (0.12–1.58)	0.167
miR205-5p	0.43 8(0.11–34.06)	0.39 (0.09–3.36)	0.73 (0.31–1.43)	0.805
miR34a-5p	0.003(0.002–0.03)	0.004(0.001–0.01)	0.003(0.001–0.02)	0.785
miR145-5p	1.49 (0.40–4.88)	1.59 (0.50–3.76)	0.42 (0.16–1.78)	0.197
miR550a	0.98 (0.42–2.65)	0.73 (0.47–1.18)	0.30 (0.17–0.98)	**0.024**

## Discussion

Our study aimed to evaluate the expression profiles of 9 EV-derived miRNAs (miR-16, miR-141, miR-10b, miR-373, miR-409, miR-34a, miR-205, miR-145, and miR-550a). The data we obtained will be discussed in this section.

miR-16-1 has been shown to target Wnt3a, which encodes CCND1 and BCL2, involved in various aspects of PCa tumor formation, including cell proliferation, survival, and invasion. Downregulation of miR-16 has been shown to lead to prostate hyperplasia associated with upregulation of CCND1 and Wnt3a; therefore, it has been determined that downregulation of this miRNA may lead to PCa development. Studies have shown that miR-16-5p is significantly reduced in PCa cells and that miR-16-5p can inhibit PCa cell survival. miR-16 functions as a major tumor suppressor targeting BCL-2 and CDK6. Its depletion facilitates activation of the PI3K/AKT survival axis [18–20].

In contrast to this study, in our study, miR-16-5p expression was found to be increased in the cancer group compared to the non-cancerous group. Interestingly, however, another study indicated that miRNA-16 in urinary EVs could be an effective and reliable biomarker for prostate cancer screening [21]. Although the observed increase in EV-derived miR-16 levels partially contradicts data supporting the role of miR-16 as a tumor suppressor in previous studies, it may reveal the potential of its EV-derived form in particular to modulate the microenvironment and direct osteoblastogenesis.

Prostate cancer tissues and cells were discovered to have higher levels of miR-141-3p than non-tumorigenic prostate tissues and cells [22]. EV-derived miR-141-3p derived from PCa cells has been shown to promote osteoblast activity. Mice injected with miR-141-3p mimicking EVs also developed significant osteoblastic bone metastasis [23]. Numerous studies have found that serum miR 141-3p levels are significantly elevated in metastatic PCa patients.

According to a different study, bone metastatic PCa tissues had lower levels of miR-141-3p expression than bone non-metastatic PCa tissues. Additionally, in vitro, overexpression of miR-141-3p inhibited PCa cell invasion, EMT, and migration [24].

The literature provides evidence that miR-141 plays a role in both the development and progression of prostate cancer and is generally upregulated. However, some studies have shown that increased miR-141-3p can suppress EMT, cell migration, and invasion. In our study, miR-141-3p expression was decreased in both BPH and localized prostate cancer (LPCa) patients compared to the control group, while no significant difference was found in the metastatic prostate cancer (MPCa) group compared to the control group. The decrease in BPH suggests that early molecular remodeling or proliferative changes may affect miR-141 levels even in benign prostatic tissue. The decrease in LPCa can be interpreted as a reflection of EMT-related changes during early local tumor development; however, the return of miR-141 expression to normal levels in the metastatic stage indicates that this effect is no longer dominant in the advanced stage [25].

miR-10b expression in breast cancer has a negative correlation with estrogen and progesterone receptor positivity and a positive correlation with HER2 positivity, regardless of metastatic status. Given that HER2 positivity and hormone receptor negativity are known indicators of tumor aggressiveness, this relationship strengthens the link between miR-10b and susceptibility to breast cancer metastases [26, 27]. In colorectal cancer, higher expression of miR-10b has been associated with advanced stage and lower survival rate [28, 29].

In contrast to these studies, another study found that miR-10b expression levels were decreased in paired liver metastases compared to primary colorectal cancer tumors [30].

In a profiling study of EVs released from PC-3 prostate cancer cells, it was determined that EV-derived miR-10b was only present in cells and not in EVs [31]. This study was conducted using cell culture, but since there is no study directly evaluating EV-derived miR-10b expression in prostate cancer patients, our study is the first to address this aspect of miR-10b. In our study, miR-10b-5p expression differed between BPH and MPCa groups and was downregulated in the MPCa group. In parallel, in the evaluation between cancer and non-cancer groups, miR-10b was determined to be down-regulated in the cancer group.

In the literature, miR-10b is generally identified as an oncogene that triggers metastasis in malignancies such as breast cancer and colorectal cancer. Its correlation with HER2 positivity, especially in breast cancer, supports the association of this microRNA with an aggressive phenotype. However, the down-regulation obtained in prostate cancer compared to the control group and in the metastatic prostate cancer (MPCa) group in our study suggests that miR-10b may not function through the same mechanism in every type of cancer. In another colorectal cancer study [30], it was shown that the level of miR-10b decreased during the transition from the primary tumor to the metastatic focus. This suggests that while miR-10b plays a role in initiating metastasis, its expression may be suppressed during the establishment and growth phase of the metastatic focus (dynamic regulation). The low miR-10b levels we observed in the MPCa group in our study are consistent with these findings, indicating a functional loss of this microRNA in the advanced stages of prostate cancer.

In PCa, like most human cancers, dysregulated CD44 expression is observed. In prostate cancer, CD44 expression found in benign epithelium is lost. It is assumed that miR-373 regulates CD44. In the study by Yang et al., miR-373 expression decreased in PCa tissues and cell lines in proportion to the decrease in total CD44 mRNA. At the protein level, miR-373 was determined to suppress CD44. Since CD44 is a tumor suppressor, miR-373 was determined to act as an oncomiR in prostate cancer [32].

However, another study shown that the level of miR-373-3p in PCa tissues was substantially lower than in BPH tissues. Furthermore, PCa patients with higher miR-373-3p expression were determined to have substantially longer overall survival (OS) than those with lower expression. Overexpression of miR-373-3p in PCa cells (LNCaP and PC3 cells) was found to inhibit cell proliferation [33].

Although the oncogenic role of miR-373 has been emphasized in many studies, its role in cancer development appears to be bidirectional. In fact, as can be seen in the two studies presented above, one study showed that it supports migration and invasion in prostate cancer, while the other found that overexpression of miR-373-3p in PCa cells substantially inhibits cell proliferation. It even has contradictory roles in prostate cancer. In our study, no difference was detected in mir-373-3p expression between the study groups.

The absence of a significant change in EV-derived miR-373 levels in our study, despite its known regulatory role (CD44 adhesion molecule) [34], may reflect the context-dependent nature of this miRNA. miR-373 is increasingly recognized as a two-faced molecule, acting as either an oncomiR or tumor suppressor depending on the specific stage of progression and cellular context. While targeting CD44 to promote invasion in specific environments, evidence suggests that miR-373 expression is dynamically regulated by hypoxia and methylation status in the tumor microenvironment. Therefore, the stable levels observed in circulation may indicate that miR-373 dysregulation is largely confined to the tissue level, or that it acts as a transient ‘switch’ in the early stages of EMT rather than a persistent systemic marker in established disease [35].

In a miRNA profiling study, stromal fibroblast-derived miR-409 was found to induce epithelial mesenchymal transition (EMT) of the epithelium adjacent to the tumor. Increased miR-409-3p/-5p expression was determined in bone metastatic prostate cancer cell lines [36]. It is stated in the literature that miR-409 is generally up-regulated in both the cancer development and metastatic processes. However, in our study, no important difference was detected when EV-derived miR-409-3p expression levels were compared between the groups.

Although miR-409 is known as a miRNA that promotes EMT and bone metastasis, the absence of changes in its circulating EV-derived levels in our study suggests that the effect occurs largely locally and does not involve systemic dissemination. miR-409-3p/-5p has been shown to promote tumor formation, epithelial-mesenchymal transition (EMT), and bone metastasis in human prostate cancer models, thus playing a significant role in modulating the tumor microenvironment [36]. Similarly, it has been reported that extracellular vesicle -borne miRNAs can affect neighboring cells through paracrine effects, and EVs released from colon cancer cells can modulate recipient cell behavior by carrying functional passenger-strand miRNAs [37]. However, stromal-enriched EVs can be significantly diluted due to the predominantly hematopoietic-derived EVs in peripheral blood; This situation makes it difficult to detect these EVs unless enriched with specific surface markers.

miR-205-5p has been found to be up-regulated in bladder and prostate cancer patients but is not involved in the inflammatory processes responsible for the BPH phenotype [38]. Additionally, miR-205-5p was determined to be up-regulated in lung cancer tissues [39].

Although the oncogenic role of miR-205 is emphasized in the literature, there are studies examining circulating miRNAs. Some miRNAs may be released into the extracellular space, but they may not participate in the process of transforming recipient cells by being packaged into EVs during the carcinogenesis process. Our study results are consistent with this, suggesting that EV-derived miR-205 is not effective in the development and progression of BPH and prostate cancer. No difference was determined in EV-derived miR-205-5p expression between the groups.

The observation that EV-derived miR-205 levels remained unchanged in our study, despite its widely documented downregulation in prostate cancer tissues, highlights the phenomenon of phenotypic heterogeneity. The fact that we did not detect a systemic reduction suggests that the majority of the circulating EVs may originate from tumor sub-clones that still retain their epithelial architecture, or that the localized loss of miR-205 at the invasive front is masked by the bulk release from non-invasive cells [40].

Levels of miR-34a-5p were down-regulated in EVs in oral squamous cell carcinoma (OSCC) patients [41]. In parallel, another study determined that miR-34a-5p was substantially down-regulated in EVs obtained from prostate cancer patients [42]. In our study, no substantial difference was determined between the groups in terms of EV-derived miR-34a-5p expression.

The lack of significant downregulation in EV-derived miR-34a levels in our cohort may be related to the specific disease stages of the patients included. Although miR-34a is a tumor suppressor often linked to the TP53 status [43]. TP53 alterations are typically more frequent in advanced castration-resistant stages rather than early-stage prostate cancer [44]. Therefore, the stable levels of miR-34a observed in our study might reflect the clinical profile of our patient group, suggesting that EV-associated miR-34a may serve as a biomarker for late-stage progression rather than an early diagnostic indicator.

EV-derived miRNA array analysis was performed to examine the effect of adipose-derived stromal cells (ASCs) on metastatic PCa. It was determined that miR-145 mediates the inhibitory effect of ASCs on prostate cancer by inducing apoptosis [45]. In our study, no substantial difference was determined in mir-145 expression between patient and control individuals.

In our study, the absence of a significant change in EV-derived miR-145 levels, despite its well-known downregulation in prostate cancer tissues, may point to a systemic masking effect. miR-145 is widely found in the human body and is highly expressed in vascular smooth muscle cells and hematopoietic cells; these cells contribute significantly to the total EV pool in circulation. The localized downregulation of miR-145 in the prostate tumor microenvironment could potentially be statistically diluted by high background levels from non-malignant systemic sources. These findings may suggest that although miR-145 is a potent tissue biomarker, its use in liquid biopsy may be limited without organ-specific EV enrichment strategies.

The significant downregulation of miR-550a-3p in the cancer group compared to the non-cancer group, and especially in the MPCa cohort compared to the BPH group, in our study suggests that this microRNA may play a role as a tumor suppressor in prostate cancer progression. Literature has shown that miR-550a-3p directly targets and suppresses ERK1 and ERK2 proteins, key effectors of the Ras/ERK pathway, in breast cancer models. These studies have demonstrated that a decrease in miR-550a-3p levels hyperactivates the ERK/RSK cascade, increasing the viability, invasion, and metastatic ability of tumor cells [46]. The decrease in miR-550a observed in the metastatic prostate cancer group in our study is consistent with the hypothesis that these oncogenic pathways may be derepressed in prostate cancer through a similar mechanism.

Some studies on lung cancer EVs have reported high levels of miR-550a in patients with brain metastases, attributing an oncogenic role to this molecule [47]. However, our findings and current studies on breast cancer show that miR-550a can also act as a tumor suppressor with the opposite effect in prostate and breast tissue. These differences may highlight that miR-550a may have a dynamic role in its function, varying depending on the cancer type and cellular microenvironment.

Although specific expression patterns were identified for miRNAs such as miR-16 and miR-550a in our study, no statistically significant difference was observed between groups for several other candidates (miR-373, miR-409, miR-205, miR-34a, miR-145). This discrepancy with tissue-based studies in the literature may be attributed to the selective sorting mechanism of EVs. Specific RNA-binding proteins (RBPs), such as hnRNPA2B1, control the loading of other miRNAs onto EVs while retaining certain miRNAs in the cell. Therefore, the lack of significant upregulation of known oncomiRs in our EV-derived samples may suggest that these miRNAs are preferentially retained in tumor tissue rather than released into circulation at the specific disease stages we analyzed [48].

PSA testing and tissue biopsy are the two main diagnostic techniques used today for prostate cancer. These two detection techniques, however, are insufficiently sensitive to identify some aggressive tumors and PCa in its early stages. Furthermore, normal PSA numbers cannot rule out PCa, and the majority of abnormal PSA values are false-positive results that could be brought on by BPH, prostatitis, or cystitis [49]. Furthermore, several biomarkers are required for the clinical diagnosis of PCa because it is a diverse and multifocal illness. As a result, minimally invasive liquid biopsies are currently being used by researchers. Extracellular vesicles are among the several components that are characterized by liquid biopsies, which examine bodily fluids [1, 50]. For these reasons, the EV-derived miRNAs evaluated in our study hold potential as specific biomarkers for prostate cancer.

The clinical value of this study may extend beyond the individual expression levels of the evaluated miRNAs, potentially highlighting a paradigm shift towards multiparameter liquid biopsies in prostate cancer management. While PSA remains the standard screening tool, its limited ability to differentiate between slowly progressing and aggressive disease can sometimes contribute to overtreatment. Our findings suggest that an EV-derived miRNA panel integrating markers such as miR-16 and miR-141 could potentially serve as a molecular reflex test for patients in the PSA gray zone. Recently, it has been emphasized that the future of oncology lies not in a single biomarker, but in the integration of stable, non-invasive molecular signatures that reflect real-time tumor dynamics. Therefore, this EV-derived panel has the potential to function as a complementary diagnostic tool, possibly helping to bridge the gap between non-specific serum screening and invasive tissue biopsies, and contributing to the reduction of unnecessary interventions in the future.

## Conclusions

Our findings suggest that specific EV-derived miRNAs may exhibit distinct expression patterns among control, BPH, and prostate cancer groups, indicating their possible role as minimally invasive biomarkers for differential diagnosis. Consistent with current research on the diagnostic relevance of EV-derived miRNAs in prostate pathology, these results provide supportive evidence for their potential value in early detection and disease stratification. Further validation in larger cohorts remains necessary to confirm their translational potential in clinical settings.

### Limitations

This study is subject to several limitations that should be considered. First, the sample size is relatively small, especially in the metastatic subgroup; this may reduce the statistical power to detect subtle differences in the expression of some miRNAs. Second, although we used a validated commercial kit for EV isolation, Western blot was not performed for EV surface markers. However, electron microscopy (TEM) was used for morphological characterization. Third, the lack of paired tissue samples prevented a direct comparison between tissue and EV-derived miRNA levels in the same patients. Future studies with larger cohorts and paired tissue-liquid biopsy designs are needed to validate the specific sequencing mechanisms of these miRNAs. Also in this study, U6 snRNA was used for normalization due to its high stability. However, we acknowledge that the use of nuclear RNAs in EV research is an ongoing subject of debate. While our results are internally consistent, the lack of a universal normalization standard in EV research remains a challenge, and future studies should consider the inclusion of exogenous synthetic spike-ins to complement endogenous controls. Also, Several comparisons demonstrated borderline statistical significance and high variability. These findings should therefore be interpreted with caution and considered hypothesis-generating rather than definitive.

## Data Availability

The datasets used or analysed during the current study are available from the corresponding author on reasonable request. Clinical trial number: not applicable.
